# Disseminated Tuberculosis Presenting as Baker's Cyst Infection

**DOI:** 10.1155/2017/6527675

**Published:** 2017-01-10

**Authors:** Najwa Pervin, Sami Akram, Tamer Hudali, Mukul Bhattarai, Sana Waqar

**Affiliations:** ^1^Department of Internal Medicine, Southern Illinois University School of Medicine, Springfield, IL, USA; ^2^Department of Internal Medicine, Division of Infectious Disease, Southern Illinois University School of Medicine, Springfield, IL, USA

## Abstract

In the absence of coexisting immunocompromised state and lack of specific symptoms a reactivation of treated mycobacterial tuberculosis (MTB) infection is generally not considered in the differential diagnosis of leg pain. We present a unique case of disseminated tuberculosis presenting as an infected Baker's cyst in a 73-year-old immunocompetent male.

## 1. Introduction

Tuberculosis (TB) can virtually affect any and all organ systems; it has also historically been known as a great mimicker; these facts coupled with the knowledge that disease course may be indolent make accurate and timely diagnosis a challenge. A high degree of vigilance is required to be able to diagnose atypical cases. TB of the appendicular skeleton is an uncommon infection caused by Mycobacterium tuberculosis and constitutes 1%–3% of all forms of TB [1]. 30% of skeletal TB involves the joints, the knee being the third most commonly affected site [2]. Bakers cyst infection with mycobacterium tuberculosis (MTB) is relatively rare and only a few cases have been described in the English literature [3, 4].

Disseminated tuberculosis is a contagious bacterial infection in which MTB bacilli spread from the lungs to other parts of the body through the blood or lymphatic system [5, 6].

Our extensive review of English literature did not yield any reports of disseminated TB presenting as a Baker's cyst infection, and this is the first reported case to the best of our knowledge.

## 2. Case Presentation

**Figure 1:**
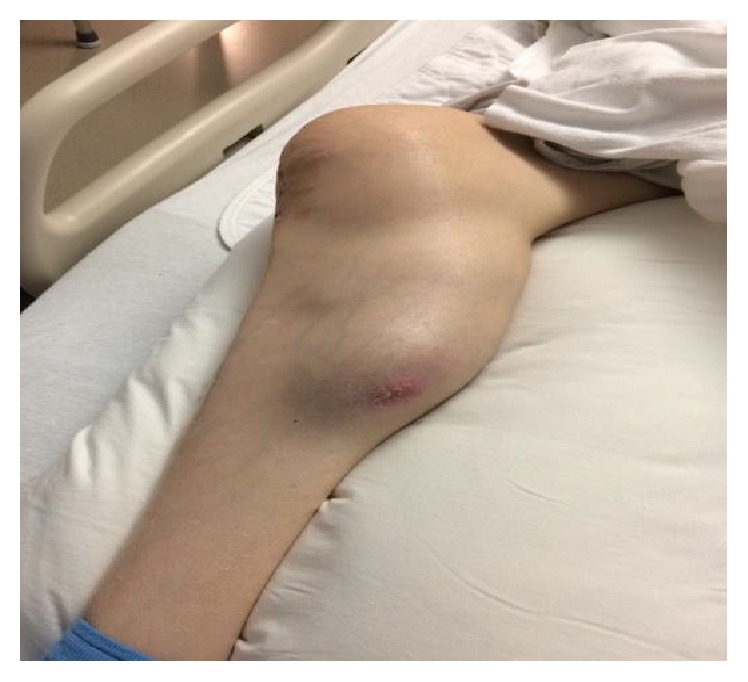
Patient's Right leg at presentation. Erythema and swelling are noticeable on the medial aspect.

**Figure 2:**
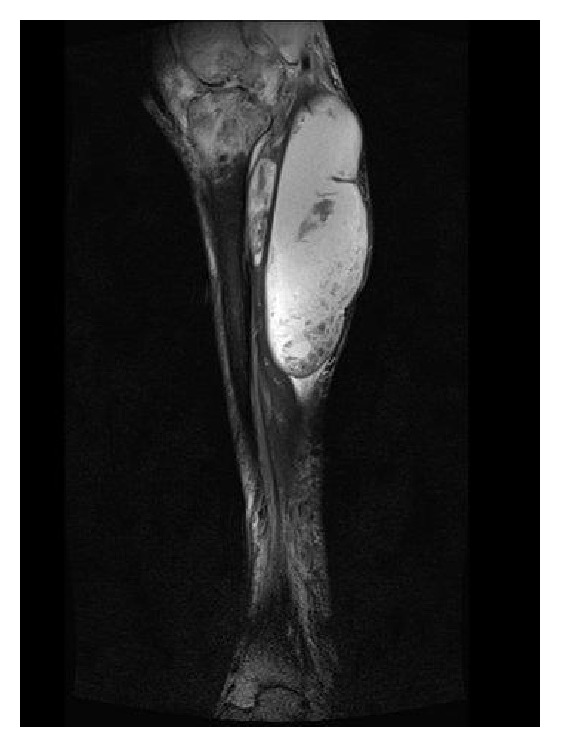
Sagittal T2-1 image from magnetic resonance imaging of right leg.

A 73-year-old Caucasian male with past medical history of Pott's disease as a child, osteoarthritis of the right knee, and a right Baker's cyst presented to the Emergency department with complaints of worsening leg pain for two days and confusion for one day. At presentation, the patient was tachycardic. All other vital signs were within normal limits. His review of systems was remarkable for chronic knee pain for the last two years and right leg pain for the last three months and decreased appetite and weight loss over the course of four months. On physical examination, the patient was in moderate distress. He was alert and oriented to time, place, and person but had poor concentration. On musculoskeletal exam there was a diffuse area of swelling on the right lower extremity which was extremely tender to touch with minimal warmth and erythema. This was located posteromedially involving the calf and medial aspect of the leg. Knee joint was nonswollen, nontender, and without any erythema or warmth (Figure 1). Laboratory data revealed leucocyte count 14.6 k/cumm, Hemoglobin 10.2 mg/dL, and hematocrit 30.4%. HIV testing was negative. His metabolic panel showed hyponatremia, 124 mEq, but was otherwise unremarkable. Leg MRI was performed. This showed a large heterogeneous fluid collection involving the subcutaneous soft tissues of the posterior medial right lower extremity measuring approximately 21.1 × 7.2 × 8.5 cm with marked mass effect on the gastrocnemius muscle. Heterogeneity of the distal femur including the femoral condyles, tibial plateau, and proximal fibular head was also visualized suggesting possible osteomyelitis as well (Figure 2). The patient underwent surgical resection of the left Baker's cyst. Intraoperative findings included a thick, boggy synovium with dull and dusky appearing cartilage. Tissue and fluid stain revealed acid fast bacillus (AFB) in one of two samples which was later identified as MTB. On pathologic section, a granulomatous reaction with giant cells was present. The patient was placed in airborne isolation and a workup for dissemination was pursued. Respiratory samples also had AFB identified on initial acid fast staining. Chest CT showed multiple apical cavitary lesions and nodular infiltrates with tree in bud opacities in the apices and right lower lobe. Acute kyphotic deformity of the upper thoracic spine with loss of vertebral body height, vertebral body fusion, posterior element fusion, and kyphosis consistent with the patient's known history of Potts disease was also seen in chest CT. Due to worsening mental status, a diagnostic lumbar puncture was also performed. CSF analysis showed 1 red blood cell/UL, 151 white blood cells/UL, 64% neutrophils, protein 1668 mg/dL, and glucose of 8 mg/dL. CSF polymerase chain reaction tested positive for MTB. Testing for HIV, Hepatitis B, and C was negative. Treatment with rifampicin, ethambutol, isoniazid, pyrazinamide, and dexamethasone was started. During the course of therapy, patient developed pneumonia and hypoxic respiratory failure requiring intubation. Family chose to proceed with comfort measures only and the patient passed away due to septic shock.

## 3. Discussion

The national TB statistics reported by the Centers for Disease Control and Prevention show that in 2014 a total of 9,421 new TB cases were reported in the United States [7]. This was a decline of 1.5% from 2013. The reported case rate was 3.0 cases per 100,000 population, which is 2.2% lower than the rate in 2013 [7]. These are the lowest rates recorded since national reporting began in 1953, although the declines are small [7]. These statistics indicate that even though the rates of TB infection are downtrending this infection is still a potential health concern.

Skeletal TB accounts for about 10–35% of cases of extrapulmonary TB and overall approximately 2% cases of TB [8]. Joints that support greater weight loads, such as the knees and the hips, are the most affected in cases of skeletal tuberculosis [9]. Peripheral osteoarticular tuberculosis is the term used for extraspinal skeletal TB that affects joints or bones [10]. Osteoarticular TB is a slowly progressive chronic disease that presents as a monoarthritis, usually of the hip or knee, in 90% of cases [11]. The osteoarticular type of TB typically results from a direct hematogenous spread of TB bacilli from the primary focus; this is mostly seen in immunocompromised individuals [12]. The rich vascular supply of large joints make this a favorable site for inoculation. Accurate diagnosis of tuberculous arthritis, especially in young, immunocompetent patients, is difficult due to its rare incidence, atypical clinical presentation, and nonspecific radiological findings [13]. Radiography may initially show soft tissue swelling but later osteopenia, periosteal thickening, and periarticular bone destruction are observed [10]. Tuberculous arthritis can often be confused with osteoarthritis, Rheumatoid arthritis, pyogenic arthritis, bone tumors, or pigmented villonodular synovitis. Tuberculous arthritis is usually characterized by synovitis and granulation exudates [12]. Classical presentation of knee tuberculosis is chronic pain, diffuse joint swelling, local tenderness, warmth, and progressive loss of function. Cold abscesses, sinuses, and constitutional symptoms are also common features. Baker's cyst is a rather rare complication of knee joint tuberculosis [4]. In fact only few cases of tubercular arthritis involving the Baker's cyst are described in literature till date [4, 14, 15].

Disseminated tuberculosis is rare in developed countries but has a strong correlation with widespread use of immunosuppressive drugs, the HIV status, and immigration patterns. Other risk factors include underlying cancer, alcohol abuse, renal failure, and diabetes [5, 6]. Early recognition and management are key to potentially improve outcomes [16]. The clinical presentation of disseminated TB is highly variable; manifestations can be acute but are more likely to be subacute or chronic. Acute disease may be fulminant, including multiorgan system failure, a syndrome of septic shock, and acute respiratory distress syndrome [17]. However, failure to thrive, fever of unknown origin, or dysfunction of one or more organ systems are presentation of chronic or subacute form of disseminated tuberculosis which are more common than the acute form [18]. The diagnostic process usually involves invasive procedures such as bone marrow and liver biopsies which are usually not obtained due to the critical conditions of the patients such as our patient, despite having a relatively high diagnostic sensitivities for disseminated TB [5, 6]. Disseminated TB can be easily missed and diagnosed only postmortem as demonstrated in recent review obtained from autopsy database [19].

In our patient, who was immunocompetent, left knee pain had started 2 years prior to presentation. He had adequate contact with health care providers and was treated for osteoarthritis with pain management and steroid injections. The absence of swelling and effusion did not alarm physicians for septic arthritis until routine cultures grew acid fast bacilli. A remote history of Pott's disease treated adequately as a child and his immune-competent state did not raise suspicion for a reactivated TB infection in his knee. The absence of any other symptoms like cough or hemoptysis further limited a timely diagnosis. This was confirmed on review of medical records from primary care physician that did not have any evidence of such symptoms during treatment for presumed osteoarthritis.

Our case serves to highlight the importance of considering a reactivated tuberculosis infection in individuals with previous history of TB even in the absence of immunocompromised state, especially if symptoms are not classic or fail to respond to conventional therapy. Delayed treatment can result in joint deformation, cartilage destruction, and even dissemination resulting in significant morbidity and mortality.
